# Establishing the Association Between Snus Use and Mental Health Problems: A Study of Norwegian College and University Students

**DOI:** 10.1093/ntr/ntac208

**Published:** 2022-08-29

**Authors:** Tore Tjora, Jens Christoffer Skogen, Børge Sivertsen

**Affiliations:** Department of Social Studies, Faculty of Social Sciences, University of Stavanger, Stavanger, Norway; Department of Health Promotion, Norwegian Institute of Public Health, Bergen, Norway; Alcohol and Drug Research Western Norway, Stavanger University Hospital, Stavanger, Norway; Centre for Evaluation of Public Health Measures, Norwegian Institute of Public Health, Oslo, Norway; Department of Health Promotion, Norwegian Institute of Public Health, Bergen, Norway; Department of Research and Innovation, Helse Fonna HF, Haugesund, Norway; Department of Mental Health, Norwegian University of Science and Technology, Trondheim, Norway

## Abstract

**Introduction:**

Smoking and mental health problems are public health concerns worldwide. Studies on smoke-free tobacco products, especially snus are scarce. Snus is considered less harmful than smoking and in the United States allowed to be marketed accordingly, but may still add to the burden of disease.

**Aims and Methods:**

Data stem from the Norwegian Students’ Health and Wellbeing Study (SHoT study) in 2018 (162 512 invited, 50 054 (30.8%) completed). Smoking, snus use, health service and medication usage and mental health problems, including the Hopkins Symptom Checklist-25 (HSCL-25), were assessed using self-report. The aims were to explore the associations between smoking and snus use and mental health problems and treatments. Furthermore, the association between both daily smoking and daily snus use and mental health problems. Associations were tested with *χ*^2^-, *t*-tests, and logistic regression.

**Results:**

Daily snus users had 38% increased odds (odds ratio [OR]: 1.38, CI: 1.30 to 1.46), and daily smokers had 96% increased odds (OR: 1.96, CI: 1.65 to 2.34) of having a high HSCL-25 score, adjusted for gender, low socioeconomic status (SES), using tobacco, participating in therapy and using antidepressants daily.

**Conclusions:**

Both daily smoking and daily snus use were associated with an increased level of mental health problems. The adjusted probability for mental health problems was lower for snus use; however, snus use prevalence was tenfold in our sample.

**Implications:**

Despite the lack of causal and directional conclusions, these associations may have implications for future legislation on snus. They also highlight the importance of more research, especially as snus is considered less harmful and seemingly replacing smoking in Norway.

## Introduction

Snus is commonly considered^1^ and marketed as a less harmful alternative to cigarettes.^1–5^ Compared with smoking, snus use is less common worldwide but has been used in Scandinavia since the early 1900s.^6,7^ In Norway, where data for this study originate, snus use has been common for a long time, but the prevalence has been rapidly increasing over the last two decades.^8^ Although Norwegian snus users were previously mainly derived from former smokers,^9^ there are newer studies showing that the majority of Norwegian students’ snus users start directly on snus.^10–12^ With the exception of Sweden, snus has been banned within the EU since 1992,^13^ and with the exception of Sweden and Finland,^14^ the prevalence is relatively low.^15^ Outside of Europe, snus is also banned in Australia^16^ and New Zealand.^17^ Despite different forms of smokeless tobacco products being sold in the United States for many years, snus was introduced as late as 2010.^18^ Snus products vary somewhat from those in Scandinavia, but the prevalence of smokeless tobacco use remains low compared with smoking.^18^ In Canada, the prevalence of smokeless tobacco products among adults was as low as 1% in 2013 and 2015.^19^ Hence, despite a long history in Scandinavia and growing interest from other countries, snus in various forms and flavors is still considered relatively unknown and new compared with cigarettes.

As the worldwide population grows, the total number of smokers also increases, despite an overall lower prevalence.^20^ Tobacco is still considered a leading risk factor for premature mortality and disability.^21^ Depression is, along with smoking, a major contributor to disease burden and disability.^22^ Several studies and reviews have demonstrated a close link between smoking and depression.^23,24^ The association appears to be bidirectional^25^ and is established in early adolescence.^26^ Similar to depression, a bidirectional association has also been shown to exist between anxiety disorders and smoking.^27–29^ Overall, it is well documented that there is at least an association between smoking and mental health problems in general, as well as specific mental disorders such as depression. These findings are supported by studies with cross-sectional designs,^30,31^ longitudinal designs,^32,33^ and time-series designs.^34^ However, all these findings have been criticized for not examining the frequency of use^35^; hence, more research on the association between smoking and mental health problems is still needed, despite the overall mentioned well-documented association between smoking and various mental health problems.

In contrast, studies on the association between smokeless tobacco in general and mental health problems remain scarce. The few existing studies have suggested a link between the use of smokeless tobacco products in general and both anxiety and depression,^36^ as well as a negative effect of the use of smokeless tobacco products among those receiving anxiety treatment.^37^ Studies specifically exploring the association between snus use and various mental health problems are virtually nonexistent, with two notable exceptions. A recent longitudinal study on Swedish adolescents (13–14 years at baseline) concluded that the onset of snus use was significantly associated with depressive symptoms.^38^ Current snus use was not significantly associated with depressive symptoms.^38^ Similarly, a Swedish study from 2010 on adults (18–75 years) found no association between snus use and mental health problems.^39^ Given the general lack of studies in this field, combined with the conflicting findings, there is a clear need for more studies focusing on the potential association between snus and mental health problems. Since snus use is considered to be less harmful^1^ compared with smoking, snus may be used to reduce the burden of diseases from smoking. Snus is already legally marketed in the United States^2^ as a less harmful product.^3,4^ Combined with increasing snus use prevalence in Scandinavia, it is crucial to investigate the potential association between snus and mental disorders such as depression and anxiety and mental health problems in general.

Based on these considerations, the first aim of the current study was to explore how smoking and snus use were associated with mental health problems and various treatments for mental health problems. The second aim was to investigate whether daily smoking and daily snus use predicted higher levels of mental health problem use, both in crude models and in models adjusted for tobacco use, therapy, or antidepressant use.

## Methods

The SHoT study (Students’ Health and Wellbeing Study), on which this study is based, is a national, ongoing, survey of students enrolled in higher education in Norway. It was initiated by the three largest student welfare organizations in Norway, and to date, three online surveys on students (aged 18–35 years) in Norway have been conducted (2010, 2014, and 2018). This study used data from the latest survey in 2018. For the SHoT2018, all full-time Norwegian students pursuing higher education (both in Norway and abroad) were invited. A total of 162 512 students fulfilled the inclusion criteria, of whom 50 054 (30.8%) completed the online questionnaires between 6 February and April 5, 2018. Further details regarding the SHoT study have been published elsewhere.^40,41^

### Instruments

#### Smoking and Snus Use

Smoking and snus use was measured with one question each: “Do you smoke?” and “Do you use snus or something similar?” The response options for both included “Yes, daily,” “Yes, occasionally” and “No.” A dichotomous variable, “daily smoking,” was operationalized as smoking daily compared with occasionally smoking or not smoking. A similar dichotomous variable on “daily snus use” was constructed. A dichotomous variable on “daily dual use” (both smoking daily and using snus daily) was constructed by combining the “daily smoking” and “daily snus use” variables mentioned above.

#### Socioeconomic Status

SES was measured with one question: “During the last 12 months, have you or your household had difficulties coping with household spending, such as food, transport, or housing?” The response options included “Never,” “Rarely,” “Occasionally,” and “Often.” “Low SES,” a dichotomous variable, was defined as answering “Often” compared with all other responses.

#### Mental Health Problems

Mental health problems were measured with a Norwegian translation of the Hopkins Symptom Checklist (HSCL-25).^42^ The HSCL-25 consists of 25 items, 10 designed to screen for anxiety and 15 designed to screen for depression.^42^ All 25 questions used an ordered categorical scale with responses from 1 to 4: (“Not at all,” “A little,” “Quite a bit,” and “Extremely”). Based on previous research using the same datasets, we chose an HSCL-25 to mean above 2.00 to make a dichotomous variable indicating high levels of mental health problems.^40^ The mean HSCL-25 score was also divided into three categories (“≤ 1.75,” “> 1.75 & < 2.00,” and “> 2.00”) for investigating monotonic associations with smoking and snus use. The 1.75 cutoffs were used as other studies also use 1.75,^43,44^ and “> 2.00” was identical to “high mental health problems” used in another study on the same dataset.^40^ There has not been performed validation studies on the use of HSCL-25 compared to structured clinical interview on students.^40^

As the survey was collected online, the participants did not receive supervision or support from a mental health professional (detailed in limitation). The survey was constructed as a web-based questionnaire completed online, hence participants were first given a list of checkboxes for screening various disorders based on a predefined list adapted to fit this age cohort. The screening list was based on a similar operationalization used in previous large population-based studies (the HUNT study^45^) and included several subcategories for most disorders and conditions (not listed here). Based on these initial screening questions, participants were asked to indicate more specific disorders or conditions. For mental disorders, the list comprised the following specific disorders or group of disorders: ADHD, anxiety disorder, autism or Asperger, bipolar disorder, depression, PTSD (posttraumatic stress disorder), schizophrenia, personality disorder, eating disorder, Tourette’s syndrome, obsessive-compulsive disorder (OCD), and other. The list contained no definition of the included disorders and conditions. In the current study, only anxiety and depressive disorders were included. We constructed a dichotomous variable for depression by coding participants, marking depression as 1 and all others as 0. A similar variable was constructed for anxiety. We chose to include both self-reported anxiety and depression in combination with the HSCL-25, as we wanted to include both a measure of whether the participants define themselves as having a mental health disorder in combination with an assessment of overall symptom load (from the HSCL-25).

#### Health Care Use

Participants were also asked to “Select if you use any of the following health services:,” Response options included: “general practitioner,” “physiotherapist,” “chiropractor,” “health station or nurse,” “psychologist or psychiatrist,” “dentist,” “emergency room,” and “other treatments.” We coded participants confirming the use of either a psychologist or psychiatrist as 1 and all others as 0 in a dichotomous variable reflecting going to therapy. Participants were also asked how often over the last 4 weeks they used different medications bought in a pharmacy; we only used responses on the use of antidepressants. Response options included “Daily,” “Weekly,” “Less than weekly,” and “Not used during past four weeks.” As very few participants chose weekly (0.23%) and more seldom than weekly (0.45%), we constructed a dichotomous variable reflecting the daily use of antidepressants (4.0%) compared with all others.

#### Statistical Analyses

Statistical analyses were computed using Stata/IC 15.1 for Windows. To address our first aim, we performed several two-way crosstabulations, examining how key variables were distributed across smoking and snus use (Table 1). We tested for differences using *χ*^2^ tests. In analyses with more than two categories, we also used the Stata tabchi package to test pairwise significance in cross-tabulations. Nonsignificant cells from this pairwise test were marked with an asterisk (Table 1). We used Bonferroni correction to avoid type 1 error. To address our second aim, we constructed two logistic regression models to examine whether the independent variables daily snus use (model 1) and daily smoking (model 2) predicted the dependent variable’s high level of mental health problems (Table 2). We also adjusted the crude models for tobacco use (snus use in the smoking model, and vice versa), gender, low SES, going to a psychologist or psychiatrist, and the daily use of antidepressants (Table 2). The results from logistic regression models were presented as odds ratios with corresponding 95% confidence intervals (CIs). Finally, we used a *t*-test to check if there were significant differences between daily snus users compared with daily smokers within the group with the most symptoms of mental health problems.

**Table 1.** The Distribution of Tobacco Use, Smoking and Snus Use Across HSCL, Depression, Anxiety, and Other Predictors

		No daily use	Daily snus use	Daily smoking	Both snus and smoking daily		*N*	difference			
Gender	Female	81.4%	17.2%	1.2%	0.2%		33 600	χ^2^ = 128.22, df = 3, *p* < .001			
	Male	77.3%	20.8%	1.5%	0.4%		14 996				
		Snus use					Smoking				
		Daily	Occasional	No	*n*	Diff	Daily	Occasional	No	*n*	Diff
Gender	Female	18.4%	8.7%^*^	72.9%	34 193	χ^2^ = 96.36, df = 2, *p* < .001	1.5%	7.1%	91.4%	33 726	χ^2^ = 293.83, df = 2, *p* < .001
	Male	22.1%	8.8%^*^	69.0%	15 251		2.0%	11.6%	86.4%	15 040	
Low SES	No	18.6%	8.7%^*^	72.8%	45 650	χ^2^ = 385.49, df = 2, *p *< .001	1.4%	8.1%	90.5%	45 049	χ^2^ = 342.04, df = 2, *p* < .001
	Yes	31.2%	9.4%^*^	59.4%	3893		4.3%	14.0%	81.8%	3818	
HSCL-25, mean	≤ 1.75	17.3%	9.0%	73.8%	29 077	χ^2^ = 249.53, df = 4, *p* < .001	1.1%	6.9%	91.9%	28 678	χ^2^ = 415.85, df = 4, *p* < .001
	> 1.75 &	21.0%	8.7%^*^	70.3%	7282		1.7%^*^	9.5%	88.9%	7199	
	> 2.00	23.7%	8.2%	68.1%	13 214		2.8%	11.5%	85.7%	13 018	
HSCL-25 > 2	No	18.0%	8.9%	73.1%	36 359	χ^2^ = 199.19, df = 2, *p* < .001	1.3%	7.4%	91.3%	35 877	χ^2^ = 356.32, df = 2, *p* < .001
	Yes	23.7%	8.2%	68.1%	13 214		2.8%	11.5%	85.7%	13 018	
Depression	No	18.9%	8.8%^*^	72.3%	44 125	χ^2^ = 114.60, df = 2, *p* < .001	1.4%	7.9%	90.7%	43 512	χ^2^ = 364.22, df = 2, *p* < .001
	Yes	24.9%	8.3%^*^	66.8%	5514		3.6%	13.6%	83.8%	5447	
Anxiety	No	18.8%	8.8%^*^	72.4%^*^	44 623	χ^2^ = 142.00, df = 2, *p* < .001	1.5%	8.0%	90.5%	44 006	χ^2^ = 264.91, df = 2, *p* < .001
	Yes	25.9%	8.0%^*^	66.1%^*^	5016		3.5%	12.9%	83.6%	4953	
Treated by psychologist or psychiatrist	No	19.2%	8.7%^*^	72.1%	44 460	χ^2^ = 33.05, df = 2, *p* < .001	1.5%	8.2%	90.3%	43 842	χ^2^ = 118.49, df = 2, *p* < .001
	Yes	22.5%	8.8%^*^	68.7%	5179		2.9%	11.4%	85.7%	5117	
Taking antidepressant daily	No	19.3%	8.7%	72.0%	41 295	χ^2^ = 98.25, df = 2, *p* < .001	1.5%	8.2%	90.3%	40 865	χ^2^ = 137.25, df = 2, *p* < .001
	Yes	28.9%	6.7%	64.5%	1726		4.6%	12.2%	83.3%	1712	

^*^ = Pairwise cell test not significant (absolute adjusted residual < 1.96).

**Table 2.** HSCL-25 > 2.00 By Daily Snus Use and Daily Smoking, Crude and Adjusted Models, in Odds Ratio With 95% Confidence Intervals

		HSCL-25 > 2.00	
		Crude model	Adjusted model^*^
Daily snus use(model 1)	No	Reference	Reference
	Yes	1.41 (1.34–1.48)	1.38 (1.30–1.46)
Daily smoking(model 2)	No	Reference	Reference
	Yes	2.26 (1.97–2.60)	1.96 (1.65–2.34)

Values are odds ratios (95% CI).

^*^ Adjusted for snus or smoking, gender, low SES, going to psychologist or psychiatrist and using antidepressants daily.

## Results

The prevalence of daily snus use was more than 10 times the prevalence of daily smoking. For females, 17.2% used snus daily, compared with 1.2% who smoked daily (Table 1). For males, 20.8% used snus daily, compared with 1.5% who smoked daily (Table 1). Males had a higher prevalence of tobacco use, both daily snus use, daily smoking, and daily use of both snus and smoking (*p* < .001) (Table 1). Males also more frequently reported occasional smoking (11.6%) than females (7.1%). However, regarding snus use, there were no gender differences: 8.8% of the males used snus occasionally compared with 8.7% of females. Participants with low SES had a higher prevalence of daily snus use (31.2%) than those whose SES was not low (18.6%). Similar to daily smoking, those with low SES had a 4.3% smoking prevalence compared with 1.4% for nonlow SES. There was no significant difference in occasional snus use regarding low SES, but low SES participants had a significantly higher prevalence of occasional smoking (14.0%) than low SES participants (8.1%).

Participants reporting an HSCL-25 mean ≤ 1.75 had a lower prevalence of daily snus use (17.3%) than participants reporting a mean > 2.00 on the HSCL-25 (23.7%, *p* < .001) (Table 1). As the pairwise test was significant, there was a monotonic association between daily snus use and the increasing HSCL mean score. Participants reporting a mean HSCL-25 ≤ 1.75 had less than half the prevalence (1.1%) of daily smoking compared with a mean HSCL-25 > 2.00 (2.8%, *p* < .001) (Table 1). In contrast to snus use, we did not find a monotonic association between daily smoking and the HSCL-25 mean score, but we did find a monotonic association between occasional smoking and the HSCL-25 mean score (Table 1).

Participants reporting having depression had a 6.0 percentage point higher prevalence of daily snus use (24.9%) than those not reporting depression (18.9%) (Table 1). Approximately 3.6% of participants who reported having depression were daily smokers, compared with 1.4% of the non-depressed participants who reported daily smoking (*p* < .001, Table 1).

Participants who reported having an anxiety disorder had a 7.1 percentage point a higher prevalence of daily snus use (25.9%) than those not reporting an anxiety disorder (18.8%) (Table 1). Participants reporting an anxiety disorder also had a higher prevalence of daily smoking, 3.5%, compared with 1.5% among participants not reporting an anxiety disorder (Table 1). In contrast to occasional snus use, occasional smoking was associated with an anxiety disorder (detailed in Table 1).

Participants reporting going to psychologists or psychiatrists had a 3.3% higher prevalence of daily snus use (22.5%) than those not reporting going to psychologists or psychiatrists (19.2%). Participants reporting going to psychologists or psychiatrists had a higher prevalence of daily smoking, 2.9%, compared with 1.5% among participants not reporting going to psychologists or psychiatrists (Table 1). In contrast to snus use, occasional smoking was associated with going to therapy (detailed in Table 1). All 16 crosstabulations on which Table 1 was based had *p*-values under .001. Therefore, the maximum possible combined *p*-value was .016. Hence, the chance of a type 1 error based on the comparison of percentages was lower than .05.

Participants reporting going to therapy had a 3.3% higher prevalence of daily snus use (22.5%) than those not going to therapy (19.2%). Participants using antidepressants daily had a higher prevalence of daily smoking, 4.6%, compared with 1.5% among participants not using antidepressants daily (Table 1). In contrast to all other predictors, occasional snus use was negatively associated with using antidepressants daily: Participants using antidepressants daily reported a lower prevalence of occasional snus use (6.7%) compared with participants not reporting using antidepressants daily (8.7%, Table 1). Participants using antidepressants daily had a higher prevalence of occasional smoking (12.2%) than those not using antidepressants daily (8.2%, Table 1).

Results from the logistic regression model predicting mental health problems showed that participants using snus daily a had 41% increased odds of having an HSCL-25 score over 2.0 (OR = 1.41, CI = 1.30 to 1.46) (Table 2). After adjusting for daily smoking, gender, low SES, going to therapy, and using antidepressants daily, participants had 38% increased odds of having an HSCL-25 score over 2.0 (OR = 1.38, CI = 1.30 to 1.46) (Table 2).

Results from the logistic regression model predicting mental health problems showed that participants who smoking daily had a 126% increased odds of having an HSCL-25 mean score > 2.00 (OR = 2.26, CI = 1.97 to 2.60, Table 2). After adjusting for daily snus use, gender, low SES, going to therapy and using antidepressants daily, participants who smoked daily had 96% increased odds (OR = 1.96, CI = 1.65 to 2.34) of having an HSCL-25 mean score > 2.00 (Table 2). Hence, the monotonic association was also persistent after adjusting. Within the HSCL-25 > 2.00 group, daily smokers had a significantly higher HSCL mean (mean = 2.61, SD = 0.42) than daily snus users (mean = 2.51, SD = 0.38, *t* = −4.65, *p* < .001). Hence, both daily snus use and daily smoking were associated with higher HSCL-25, and for those with HSCL-25 > 2.00, the daily smokers had a significantly higher mean.

## Discussion

The main finding of this study, based on a large sample of Norwegian college and university students, was the increased odds of mental health problems both for snus users and smokers. The increased odds were also persistent after controlling for known confounders such as gender, low SES, and various forms of treatment. Furthermore, the increased odds were also persistent after controlling for other types of tobacco use. Regarding snus use, this is highly interesting, as the finding indicates that current daily snus use was associated with higher odds of mental health problems after controlling for daily smoking. As Norwegian smoking prevalence is decreasing and most new snus users have never smoked,^10–12^ this finding indicates that snus use in itself is associated with higher prevalence of mental health problems.

Another novel finding regarding snus use was the monotonic association found in the cross-tabulation between daily snus use and mental health problems. In contrast, there was no monotonic association between daily smoking and mental health problems; however, there was no monotonic association between occasional smoking and mental health problems. Furthermore, the cross-tabulation revealed a higher prevalence of daily snus use for participants reporting depression or anxiety. Finally, we observed an association between daily snus use and receiving treatment or using antidepressants daily. Another important finding was that this study also supports the more well-documented association between smoking and mental health problems. Additionally, the study showed that occasional snus use was not associated at all with mental health problems, in contrast to occasional smoking, which was associated with mental health problems although not monotonic as mentioned above.

Our study supports well-established findings from several other studies on the association between smoking and depression,^23,24^ anxiety,^27–29^ and mental health problems.^30–34^ More novel is the reported association between snus use and depression, also supported by a recent Swedish longitudinal study.^38^ Similar to studies on smokeless tobacco in general,^36^ the present study establishes an association between snus use, anxiety, and mental health problems.

Compared with smoking, the increased odds of depression, anxiety, and mental health problems were smaller for snus use in our study, a finding also reported by the Swedish study.^38^ Furthermore, the adjusted probability for mental health problems associated with snus use is lower compared to smoking. However, given the over 10 times higher daily prevalence of snus use in the Norwegian student population, the overall potential burden of increased mental health problems may become larger because of snus use compared to smoking, especially if smoking prevalence decreases further. Although cause-effect relationships cannot be drawn based on our study findings, this finding is still important, as it highlights the potential importance of snus use in predicting and understanding mental health problems, depression, and anxiety.

Another important finding in this study was the differences between occasional snus use and occasional smoking. To the best of our knowledge, occasional snus use in association with either mental health problems, depressive or anxiety disorders have not been previously studied. Without exception, scoring high on the included predictors, such as mental health problems, reporting depression or anxiety, reporting going to a psychologist or psychiatrist, or using antidepressants daily, was associated with a significantly higher prevalence of occasional smoking. Occasional smoking becomes an intermediate position between daily smoking and nonsmoking. Occasional snus use seems different, as there were no such associations between the same predictors and occasional snus use. However, there was one notable exception, although the association appears to be in the opposite direction: reporting the use of antidepressants daily was associated with a two percentage points lower prevalence of snus use (6.7% compared with 8.7%). Participants using antidepressants daily had an almost ten percentage points higher daily prevalence of snus use, similar to smoking.

### Implications

The present study indicates that snus use is associated with anxiety, depression, and overall higher levels of mental health problems among Norwegian college and university students. This may indicate the need for anti-snus campaigns similar to anti-smoking campaigns used in many countries for decades, although the study does not show that snus use causes mental health problems. Furthermore, countries considering introducing snus to be sold as a less harmful alternative to cigarettes may reconsider, especially if smoking prevalence is low or already decreasing without snus already introduced to their markets. The finding also suggests a general association between tobacco products and both depression and anxiety, an association already found between e-cigarettes and the vaping of nicotine-containing liquids and several mental health problems.^46–48^

Compared with the smoking epidemic, the snus epidemic in Norway^49^ is in an earlier stage. Furthermore, the snus epidemic seems to develop much more quicker than smoking did, and it also appears to affect a smaller proportion of the Norwegian population as snus prevalence has not reached the same high prevalence as smoking did in the sixties and seventies.^50^ For smoking, there was a lag between increasing smoking prevalence and health problems in general^21,51^; hence, the costs of the epidemic appear more clearly in the latter stages.^51^ If snus use follows a similar, but quicker, development as smoking, this has several implications: First, it indicates that established associations between snus use and mental health problems may have emerged during the snus epidemic and were not present from the start of the epidemic. We believe that the present study supports this implication. Second, it indicates that as the snus epidemic in Norway^49^ evolves, the present increased prevalence of mental health problems in snus users will probably increase as female snus users also increases.

### Strengths and Limitations

A major limitation was the relatively modest participation rate of 31% (detailed in Methods).^40,41^ Such nonparticipation could be associated with key variables in this study, such as smoking, snus use, gender, and mental health problems. However, another study based on a very similar population showed similar findings on key variables, such as daily smoking and daily snus use.^10^ Another limitation was the use of cross-sectional data, limiting the possibility of following individual and group trajectories. We did not control for former smoking or snus use, which also is a limitation. Furthermore, the study only included self-reported measures in an online survey and included only Norwegian young adults pursuing higher education. It was also a limitation that HSCL was administered online without professionals administrating it.

The snus use question, “Do you use snus or something similar?”, measured chewing tobacco in addition to snus use. However, as only seven tons of chewing tobacco were sold compared with 1487 tons of snus in Norway in 2018,^7^ we consider this to be a minor limitation. Study strengths include one of the very few investigating the association between snus use and mental health. Other strengths; the large sample size and use of a widely used and validated questionnaire measuring mental health problems— the HSCL-25^42^—as well as self-reported anxiety and depressive disorder.

### Further Research

Few studies on the association between snus use and mental health call for replications using both similar and more general populations. As snus use in Finland is currently increasing,^52^ despite the EU ban, a similar study following the development of snus use and its association with mental health in a Finnish population would be interesting. As mentioned in the introduction, the frequency of smokeless tobacco use, especially snus use, is underexamined; hence, more studies focusing on snus use frequency in association with mental health problems are needed.

## Conclusions

This study, based on a large sample of Norwegian students, shows that there was a significant association between snus use and mental health problems, as well as self-reported depression and anxiety disorder. Both daily smoking and daily snus use were associated with a higher prevalence of mental health problems. Given the 10 times higher prevalence of daily snus use, the association between snus use and mental health problems should be taken into consideration. The study highlights the importance of continuing research on tobacco use and its association with other negative health effects, perhaps especially on snus in those markets where it is both relatively unknown and new but marketed as a less harmful alternative.

## Supplementary Material

A Contributorship Form detailing each author’s specific involvement with this content, as well as any supplementary data, are available online at https://academic.oup.com/ntr.

ntac208_suppl_Supplementary_Taxonomy-form

## Data Availability

The SHoT dataset is administered by the NIPH. Approval from a Norwegian regional committee for medical and health research ethics https://www.forskningsetikk.no/en/about-us/our-committees-and-commission/rek/ is a prerequisite. Guidelines for access to SHoT data are found at https://www.fhi.no/en/more/access-to-data
